# Assessment of Efficacy of Algerian Propolis against the Parasitic Mite *Varroa destructor* and Safety for Honey Bees by Spray Treatment

**DOI:** 10.3390/insects15010075

**Published:** 2024-01-22

**Authors:** Ahmed Sabri Ayad, Samia Benchaabane, Tarek Daas, Guy Smagghe, Wahida Loucif-Ayad

**Affiliations:** 1 Laboratory of Applied Animal Biology, Faculty of Sciences, Badji Mokhtar University, Annaba 23000, Algeria; ayad_sabri@yahoo.fr (A.S.A.); tarek63daas@yahoo.fr (T.D.); 2 Department of Plants and Crops, Faculty of Bioscience Engineering, Ghent University, 9000 Ghent, Belgium; 3 Institute of Entomology, Guizhou University, Guiyang 550025, China; 4 Department of Biology, Vrije Universiteit Brussel (VUB), 1050 Brussels, Belgium; 5 Faculty of Medicine, Badji Mokhtar University, Annaba 23000, Algeria

**Keywords:** propolis, *Varroa destructor*, acaricide activity, *Apis mellifera*, spray

## Abstract

**Simple Summary:**

*Varroa destructor* is an ectoparasitic mite that affects honey bee colonies and it is considered one of the most important causes of honey bee losses. Botanical origins have emerged as natural alternative acaricides to diminish the population levels of Varroa mites. Propolis is a natural product, consisting of a complex mixture of resinous substances collected by honey bees from different plant sources. In this study, we investigated the effect of propolis, collected by native Algerian honey bees, on *V. destructor* by a spraying method. The results indicated that propolis extracts at 10% are effective in killing Varroa mites and are harmless for honey bees. Propolis extracts could be used in honey bee colonies by spraying to control Varroa mite infestations, and further investigations are required for a better understanding of the mechanism(s) of this acaricide activity.

**Abstract:**

*Varroa destructor* is an ectoparasitic mite and is considered one of the most important causes of honey bee population loss. In the last years, substances of botanical origin have emerged as natural alternatives to diminish the mite population levels. Propolis is a natural product and is used by honey bees for multiple tasks, including protection from pathogens and parasites, and varroacidal activity of propolis extracts has been shown. In this study, we investigated the potential of propolis, collected by native Algerian honey bee subspecies (*Apis mellifera intermissa* and *A. m. sahariensis*) in different locations in Algeria and extracted by ultrasound, to control mites of *V. destructor* and tested the safety for the honey bees. The most important results were that the best propolis extracts at 10% killed 100% of the Varroa mites within 3–4 h in a Petri dish assay. In addition, when we sprayed *A. m. intermissa* bees infested with Varroa mites with a 10% concentration in a mini-hive setup, we scored a high mite mortality of 85–87% with the best propolis extracts, and importantly, there was no mortality in the bees. Our data demonstrated that propolis extracts in Algeria could be used in honey bee colonies by spraying against Varroa mite infestations, which may develop as an easy method for local beekeepers to control Varroa in their hives. Further research should investigate the mechanism of action.

## 1. Introduction

Honey bees are effective pollinators of multiple crops, and managed honey bees become increasingly important for the regulation of ecosystems [1]. In the past decade, beekeepers have been confronted with high levels of mortality in their colonies worldwide [2]. Several factors are associated with honey bee population decline, including a loss of natural habitat, climate change, the use of pesticides, and diseases caused by a large spectrum of bacteria, viruses, or fungi [3,4,5]. The parasitic mite, *Varroa destructor* [6], plays a fundamental role in the decline of honey bees. This parasite feeds on the bee’s fat body and hemolymph [7], causing a number of detrimental effects on bees at the individual level [8], and is involved in the transmission of several bee viruses [9]. Infestation with Varroa mites is generating a fatal disease epidemic within the colony and causing great economic losses to the beekeeping industry [10,11].

Various methods, including physical, biological, and chemical ones, have been applied to control the Varroa mites. Synthetic acaricides (e.g., tau-fluvalinate, flumethrin, amitraz, and coumafos) have been the traditional way of control during the last years, but they cause lethal effects on bees, and there is a build-up of chemical residues in hive products and the development of insecticide-resistant mite populations [12,13]. New natural treatments that minimize the above hazards have been developed using organic acids (formic acid, oxalic acid, and lactic acid), essential oils (thymol, carvacrol, and menthol) [3,14,15], and propolis [16,17,18,19,20] because they naturally occur in bee colonies and possess significant acaricide activities [21].

Propolis is a resinous mixture collected by honey bees from tree buds, sap flows, and other botanical sources and mixed with bee secretions. It is a fundamental substance for the sealing and sterilizing of the hives to prevent the development of microbial diseases, such *as* bacteria, viruses, and fungi [22]. The chemical composition of propolis varies depending on many factors, including the source of the plant, and generally, it is composed of 50% resins, 30% vegetable balsams, 10% wax, 5% aromatic and essential oils, 5% pollen, and other natural products [23]. Several studies have shown the biological activities of various propolis as having anticancer, anti-inflammatory, antioxidant, antimicrobial, and antifungal properties, and these interesting properties are attributed to the presence of different compounds, such as flavonoids, phenolic compounds, aromatic acids, and terpenes, which can work alone or together [24,25]. In detail, propolis is used by honey bees for multiple tasks, including protection from pathogens and parasites, but low solubility prevents some of its active components from having a direct effect; also, varroacidal activity of propolis extracts has been shown in several papers [21,22,23,24,25,26,27,28,29,30,31].

The aim of this study was to investigate the acaricide activity of propolis extracts collected by the two native Algerian honey bee subspecies. *V. destructor* mites were topically treated in a Petri dish setup. In addition, we infested honey bees with Varroa mites, sprayed them with the propolis extracts in a mini-hive setup, and followed the efficacy against the mites and the safety of the honey bees. We believe that our data contribute to the use of propolis extracts by spraying honey bee colonies to control Varroa mite infestations. For instance, local beekeepers that are confronted with high levels of mortality in their colonies can be helped by this easy approach that is efficient and safe for their bees.

## 2. Materials and Methods

### 2.1. Propolis Collection and Extraction

The propolis samples (about 30 g per location) were collected with the help of beekeepers of the National Association of Professional Beekeepers from three queen breeding stations that are located in Annaba (36°54′40.8″ N 7°41′46.5″ E), Medea (36°14′12.9″ N 2°57′22.6″ E), and Ghardaia (32°44′54.5″ N 4°31′10.9″ E), which are in the northeast, center, and south of Algeria, respectively. The local bee subspecies present at apiaries of Annaba and Medea was *Apis mellifera intermissa*, and *Apis mellifera sahariensis* was at Ghardaia. *A. m. intermissa* is a dark honey bee with a long tongue that is prone to swarming, shows aggressive behavior, and has abundant use of propolis [9]. Also, this subspecies seems to be more susceptible to Varroa mites than other African subspecies and is more affected by these mites compared to other bee species in the rest of Africa. *A. m. sahariensis* is smaller and yellowish-reddish, has a short tongue, small hairs, and a large tomentum, and is characterized by a moderate swarming tendency, little use of propolis, and a weak defense reaction [32]. The propolis samples were collected from plastic grills previously placed in the hives and stored in the dark at −20 °C until extraction.

For ultrasound extraction, we followed the protocol of [33]. At first, the propolis samples were crushed in a chilled mortar, sieved, and kept at −20 °C once powdered. Then, 5 g of powder propolis sample was added to 20 mL of 70% ethanol and placed in an ultrasound liquid processor (Sonics: Vibra-Cell VCX130 Ultrasonic Processor with Sound Abating Enclosure) for 20 min (40 W; 20% amplitude). These mixtures were kept for 24 h and then were centrifuged for 10 min at 1644× *g*. The suspensions were frozen at −20 °C for 24 h and filtered to remove waxes, and the solutions were dried under vacuum at room temperature.

### 2.2. Toxicity Bioassays with Varroa Mites and Honey Bees

**Figure 1:**
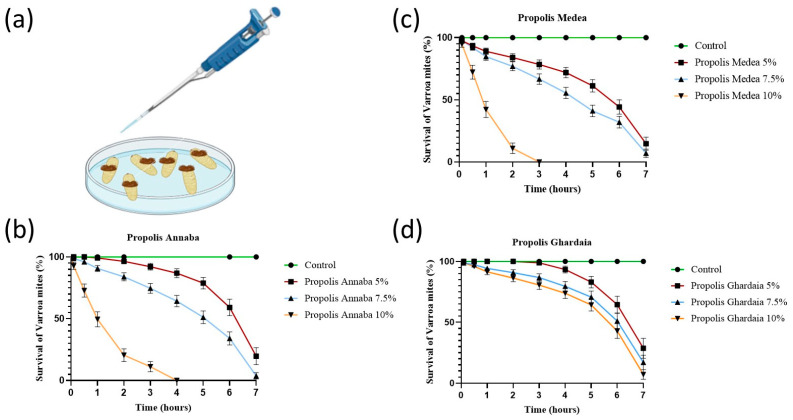
(**a**) Petri dish setup wherein *V. destructor* mites were treated by the topical method. We used *V*. *destructor* mites from honey bees (*A. m. intermissa*) that were not treated in the preceding 12 months. For the topical treatment of the mites, we placed 6 mites on a filter paper (3 × 3 cm), and then we applied 200 µL of different concentrations of the propolis extracts (prepared in 40% ethanol) per mite. The controls were treated with 200 µL of 40% ethanol. Survival rates of *V. destructor* after treatment with the three different Algerian propolis extracts from (**b**) Annaba, (**c**) Medea, and (**d**) Ghardaia at three concentrations of 5%, 7.5%, and 10% (*w*/*v*) by the topical method. The survival curves of Varroa mites were analyzed according to the Kaplan–Meier method and compared to each other by a log-rank Mantel–Cox test. We used 6 mites per experimental unit (*n* = 6), and the experiment was performed with 3 replicates, according to the protocol of [26].

In the first series of experiments, we tested the efficacy of the propolis extract against Varroa mites in a setup to score acute mortality in a Petri disc setup (Figure 1a), using the protocol of [26] with some slight modifications. We used *V*. *destructor* mites from honey bees (*A. m. intermissa*) that were not treated in the preceding 12 months. The mites were collected with a fine paintbrush and placed in a Petri dish with live honey bee larvae and pupae to prevent starvation during harvesting operations. For the topical treatment of the mites, we placed six mites on a filter paper (3 × 3 cm), and then we applied 200 µL of different concentrations of the propolis extracts per mite (Figure 1a). The different concentrations (*w*/*v*) of the dried propolis extract powder were prepared in 40% ethanol and consisted of 5%, 7.5%, and 10%. After the contact with the propolis for 30 s, the mites were placed in a new untreated Petri dish and scored for survival at 10, 30, and 60 min and then each hour for 7 h in total. Mortality was evaluated by gently prodding each mite with a narrow paintbrush; lack of response to consecutive stimulus was considered an indication of death. In the control, the topical treatment was performed with 200 µL of 40% ethanol alone. For each concentration, we performed three biological repetitions.

**Figure 2:**
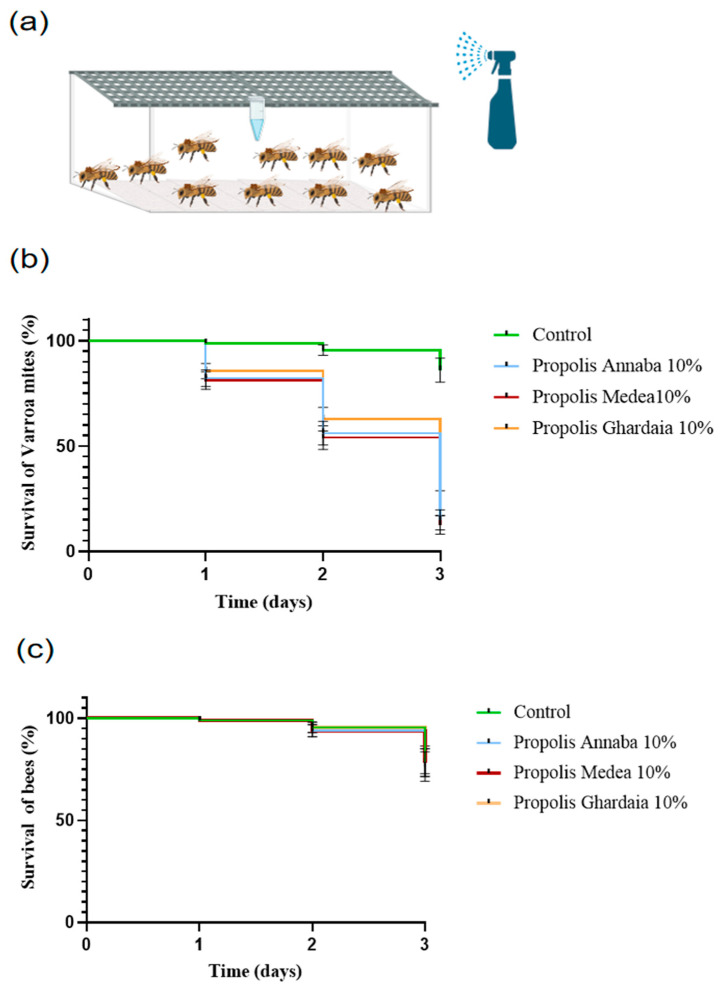
(**a**) Mini-hive setup wherein *V. destructor* mites and honey bees (*A. m. intermissa*) were treated by spraying. We placed 10 adult female mites and 10 newly emerged bee workers together in a plastic box (13 × 9 × 4 cm) that was lined with filter paper on the bottom, and a 1.5 mL Eppendorf tube provided sugar syrup to the honey bee workers. Then, once the mites were attached to the body of the honey bee in each experimental box, we sprayed 3.4 mL of the 10% propolis extract concentration (prepared in 40% ethanol) on the paper arena (with the honey bees and mites) with a hand sprayer. The controls were sprayed with 3.4 mL of 40% ethanol. Survival rates of (**b**) *V. destructor* and (**c**) honey bees (*A. m. intermissa*) after treatment with three different Algerian propolis extracts at 10% (*w*/*v*) by the spraying method. Data were analyzed according to the Kaplan–Meier method and compared to each other by a log-rank Mantel–Cox test. We used 10 mites and 10 bees per experimental unit (*n* = 10), and the experiment was performed with 3 replicates, according to the protocol of [26].

In a second experiment, we sprayed the concentration that caused 100% mite mortality in the Petri dish assay (i.e., 10%), on the Varroa mites, and on the honey bees (*A. m. intermissa*) they were feeding on in a mini-hive setup (Figure 2a) based on the protocol of [26] with some slight modifications. At first, we placed 10 adult female mites and 10 newly emerged bee workers together in a plastic box (13 × 9 × 4 cm) that was lined with filter paper on the bottom. Then, once the mites were attached to the body of the honey bee in each experimental box, we sprayed 3.4 mL of the propolis extract concentration on the paper arena (with the honey bees and mites) with a hand sprayer. The 10% concentration (*w*/*v*) of the dried propolis extract powder was prepared in 40% ethanol, as in the first experiment. For the control groups, we sprayed 10 mites and 10 bees per box with 3.4 mL of 40% ethanol. After treatment, the boxes were placed in an insect incubator in darkness at 30 °C and 70% relative humidity, and we provided sugar syrup as food to the bees. The mortality of mites and honey bees was assessed after 1, 2, and 3 days by prodding each mite or honey bee with a narrow paintbrush. Lack of response to consecutive stimulus over 1 min was considered an indication of death. Per concentration, we performed three biological repetitions.

All experiments were performed in triplicate, and the data were denoted as mean ± standard error of the mean (SEM). Survival curves of mites and honey bees were set up following the Kaplan–Meier method and compared to each other by a log-rank Mantel–Cox test. The results were analyzed with Prism 8.0.2 (GraphPad, San Diego, CA, USA).

## 3. Results

Figure 1b–d present the acaricide activity of the propolis samples from three different locations and at three different concentrations against *V. destructor* in a Petri dish setup to score acute mortality. Seven hours after topical treatment of the mites, it was clear that the Varroa mites were highly susceptible to the propolis extracts, and the percentage of mites killed by the treatment ranged between 71% and 100% over the three concentrations and the three propolis samples. The survival curves showed a significant decrease for the mites after exposure to the propolis of Annaba (log-rank test: χ^2^ = 251.8; *p* < 0.0001), Medea (χ^2^ = 317; *p* < 0.0001), and Ghardaia (χ^2^ = 39.72; *p* < 0.0001). The mortality of mites increased with an increase in concentration. The extract concentration of 10% was very effective, killing 100% of the mites with the propolis from Annaba and Medea and 92% with the propolis from Ghardaia, but this difference was not significant (*p* > 0.05). The propolis of Medea and Annaba had the strongest acaricide activity of 100% mortality of mites after only 3–4 h (*p* > 0.05). The propolis of Ghardaia caused 92% of mites to die after 7 h (*p* < 0.01).

In the second experiment, we sprayed the best concentration of the previous assay (i.e., 10%) on the Varroa mites and the honey bees they were feeding on in a mini-hive setup. In this setup, the spraying of a 10% concentration of propolis extracts from the three locations could significantly kill the mites (log-rank test χ^2^ = 65.65; *p* < 0.0001). Figure 2b shows that 10% of Annaba and Medea propolis extract caused 85% and 87% mortality in the mites at 3 days after spraying compared the control (*p* < 0.05), while Ghardaia propolis results in 77% mortality, but the difference was not significant (*p* > 0.05) compared to Annaba and Medea. On the other hand, the survival curves of the honey bees (on which the mites were feeding), after the spraying with 10% propolis extracts (Figure 2c), demonstrated that there was no loss of survival compared to the control (log-rank test: χ^2^ = 0.1002; *p* > 0.05), confirming that the three Algerian propolis extracts at 10% are harmless to honey bees. The mortality in the control honey bees that were sprayed with 3.4 mL of 40% ethanol (with no propolis extract) was also less than 25%.

## 4. Discussion

In this study, we showed that propolis extracts from Algeria can be used to control Varroa mites efficiently by spraying, and it did not kill the honey bees. Our results contribute to the search and development of new alternative strategies to control Varroa mites in order to reduce the use of and the amount of synthetic acaricides in the honey bee hives. We believe that our data will be very useful for practice, particularly for local beekeepers who are confronted with high levels of mortality in their colonies and who can use this easy method of spraying that is efficient and safe for their honey bees. In addition, it should be remarked that extracts of propolis also have extra interesting benefits to the health of honey bee hives with a remarkable action against many microorganisms, such as the causative agents of chalkbrood (*Ascosphaera apis*) [22] and American foulbrood (*Paenibacillus larvae*) [27,28].

In our study, the spraying of Varroa mites and honey bees in a mini-hive setup with 10% Algerian propolis extract killed the Varroa mites efficiently and was harmless for the bees. At present, there is unfortunately no information available on the mode of action of the propolis extracts. Based on Garedew et al. [34] and Hassan et al. [35], it is suggested that contact with the propolis solution could lead to a weakening of the mite’s cuticle, which could facilitate entry of the active compounds present in propolis. In propolis, a wide spectrum of bioactives is reported to be present; for instance, flavonoids, phenolic compounds, aromatic acids, and terpenes, and these can act synergistically [25]. However, nothing is known so far about their mechanism to kill mites. Future investigations should unravel the mechanism(s) of the high acaricide activity. Also, it should be reported that propolis may increase the honey bee’s immunity with an enhancement of its defensive response [30]. Similar results have been obtained with propolis from Germany and Argentina at a concentration of 10%, which killed 100% of Varroa mites [26,29]. The latter results, together with ours, clearly demonstrate that Varroa mites are highly susceptible to propolis and that propolis extracts cause narcosis and the death of mites after a contact treatment. The results obtained based on the contact with propolis extracts, at the concentration tested, suggest that this method should be recommended. A spray treatment is easy and practical. A treatment of propolis extract via oral intake is not recommendable since [31] found that delivering propolis via the bee syrup affected the abdominal fat bodies and the hypopharyngeal gland development of the honey bees. Also, previous research has shown that currently used acaricides and insecticides pose negative effects on honey bees of *A. m. intermissa* at the concentrations used to control Varroa mites [36,37,38]. Further research is required to identify the best concentration of propolis extract and frequency of treatment by spraying honey bee hives in practice.

Under normal conditions, the honey bee workers apply propolis on the walls of the beehive and the frames; however, the Varroa mites that are living in these hives are not affected, and no direct acaricide activity is detected. It is likely that propolis is insoluble in the interior of the bee hive since most active components of propolis are water-insoluble [31,39]. When propolis is extracted from an organic solvent, such as ethanol, the most biologically active components are obtained [39,40]. Also, the use of ultrasound technology enhanced the extraction of propolis compounds [33,40], generating extracts with higher biological activity [41]. Garedew et al. [29] and Ding et al. [42] reported that a concentration of 70% was most efficient in extracting, and this finding agreed with a high lethal activity against mites. However, the authors did not score the safety of honey bees for such high ethanol concentrations. In fact, ingestion of ethanol in honey bees can cause immune suppression [43], affect appetitive learning and olfactory perception [44], and cause mortality in a dose–dependent manner [45]. In our experiments, after drying the propolis extracts, we used 40% ethanol to reduce the effect of the strong ethanol solution and after, we used it in the spraying method to treat honey bees against Varroa mites in a safe manner. More research in extraction optimization is possible.

In this study, we investigated propolis samples from three regions in Algeria. Particularly, we used propolis from Annaba, which is from Mediterranean northern Algeria and produced by *A. m. intermissa*, which is a long-tongued bee, and propolis from Medea, which is produced by the same bee strain in a more transitional climate in the northern hills. These two regions are rich in plants and water. On the other hand, Ghardaia propolis was collected from a more southern desert region in Algeria by *A. m. sahariensis,* and the climate is dry in this region. *A. m. sahariensis* is short-tongued and adapted to the date palm (*Phoenix dactylifera*) and other Saharan flora. In parallel to this project, an LC-MS analysis of the three propolis samples [46] identified more than 20 compounds, and pinocembrin and pinobanksin-3-acetate were amongst the most abundant compounds identified in the extracts of the three locations. But, there were some differences between the extracts. Particularly, pinostrobin was more abundant in the Annaba extract than in the Medea extract, and this component was not detected in the Ghardaia propolis extract. The Ghardaia extract contained more ferulic acid compared to the other two extracts. We think that the variability between the results obtained by Algerian propolis samples, although the differences in activity were small (<20%), could be due to the difference between bee subspecies and botanical sources since previous research has demonstrated that the composition of propolis may depend on these factors [47,48]. However, future experiments with a more specific experimental design should answer these questions. In addition, we note that based on the literature, several of the identified components in the propolis samples are considered the main effective phytochemical components responsible for biological activities attributed to propolis [46,49]. As a consequence, we believe that the acaricide activity of propolis extracts is due to the bioactive components, such as phenols and flavonoids, present in propolis [19,26,50]. Future investigations should unravel the mechanism(s) behind the high acaricide activity.

## 5. Conclusions

In conclusion, our data do not only show that the spraying of a 10% concentration of propolis extract (made in 40% ethanol) was very efficient and could kill 100% of the Varroa mites in 3 days in a mini-hive setup but also that this spray treatment was safe for the honey bees. In turn, these findings suggest that propolis extracts from Algeria could be used in honey bee colonies by spraying to control Varroa mite infestations. But, further optimization on extraction, appropriate doses, and concentration to be administered is useful. Also, future research should unravel the mode of action of this high acaricide activity by propolis extracts.

## Data Availability

Dataset available on request from the authors.
